# Sarcopenia: burden and challenges for public health

**DOI:** 10.1186/2049-3258-72-45

**Published:** 2014-12-18

**Authors:** Charlotte Beaudart, René Rizzoli, Olivier Bruyère, Jean-Yves Reginster, Emmanuel Biver

**Affiliations:** Department of Public Health, Epidemiology and Health Economics, University of Liège, Avenue de l’Hôpital 3 – CHU B23, Liège, 4000 Belgium; Support Unit in Epidemiology and Biostatistics, University of Liège, Liège, Belgium; Division of Bone Diseases, Geneva University Hospitals and Faculty of Medicine, Rue Gabrielle Perret-Gentil 4, Geneva, 14 CH-1211 Switzerland; Bone, Cartilage and Muscle Metabolism Unit and Chair of the Department of Public Health Sciences, CHU of Liège, Quai Godefroid Kurth 45, Liège, 4000 Belgium; Department of Motricity Sciences, University of Liège, Liège, Belgium

**Keywords:** Sarcopenia, Public health, Epidemiology, Consequences, Diagnosis

## Abstract

Sarcopenia, operationally defined as the loss of muscle mass and muscle function, is a major health condition associated with ageing, and contributes to many components of public health at both the patient and the societal levels. Currently, no consensual definition of sarcopenia exists and therefore it is still a challenge to establish the actual prevalence of sarcopenia or to establish the direct and indirect impacts of sarcopenia on public health. Anyway, this geriatric syndrome represents a huge potential public health issue because of its multiple clinical and societal consequences. Moreover, all these aspects have an impact on healthcare costs both for the patient and the society. Therefore, the implementation of effective and broadly applicable preventive and therapeutic interventions has become a medical and societal challenge for the growing number of older persons affected by sarcopenia and its disabling complications.

**Electronic supplementary material:**

The online version of this article (doi:10.1186/2049-3258-72-45) contains supplementary material, which is available to authorized users.

## Background

Thanks to social, health and technological progress, the proportion of older people in the age pyramid is increasing all over the world. According to the World Health Organisation, in 2050 there should be at least 2 milliards of people aged 65 years or older, compared to 600 million today. The life expectancy is also increasing and is estimated around 80 years in industrial countries [1]. The aging process is responsible of many changes in body composition including a loss of skeletal muscle mass. From the age of 25, there is a progressive decrease in the size and number of muscle fibres resulting in a loss of about 30% of muscle mass at the age of 80 [2]. Beyond some defined threshold, this age-related loss of muscle mass is characterized as abnormal. To characterize this phenomenon, the term “sarcopenia” was firstly introduced by Irwin Rosenberg [3]. The definition of sarcopenia was then enriched with scientific and technological advances and gradually evolved to incorporate the notions of decreased muscle mass [4], then of decreased muscle function (low muscle strength or low physical performance) [5–11]. These definitions differ from each other in regards to muscle mass indicators (ratio of appendicular lean mass over height squared, ALM/ht^2^, or over body mass index, ALM_BMI_), the cutpoints for slow gait speed and whether or not they include a measure of weakness (Table 1). However, there is actually no universal consensus for an operational definition of sarcopenia, which is an important issue for public health.

**Table 1:** **Proposed operational definitions of sarcopenia**

Criteria		Muscle mass	Muscle function		
			Muscle strength		Physical performance
Baumgartner criteria [4]	Sarcopenia	ASM/ ht^2^ > 2 SD below young healthy mean	x		x
European Society for Clinical Nutrition and Metabolism Special Interest Groups (ESPEN-SIG) [7]	Sarcopenia	Percentage of muscle mass ≥2 SD below mean in young adults of the same sex and ethnic background (individuals aged 18–39 years in the NHANES III cohort)	x		Gait speed: <0.8 m/s or Reduced performance in any functional test used for comprehensive geriatric assessment
European Working Group on Sarcopenia in Older People (EWGSOP) [8]	Sarcopenia	ALM/ht^2^	Grip strength	OR	Gait speed: <0.8 m/s
		- Men: ≤7.23 kg/m^2^	- Men: <30 kg		
		- Women: ≤5.67 kg/m^2^	- Women: <20 kg		
	Severe sarcopenia			AND	
International Working Group on Sarcopenia (IWGS) [9]	Sarcopenia	ALM/ht^2^	x		Gait speed: <1.0 m/s
		- Men: ≤7.23 kg/m^2^			
		- Women: ≤5.67 kg/m^2^			
Society of Sarcopenia, Cachexia and Wasting Disorders [10]	Sarcopenia with limited mobility	ALM/ht^2^ > of 2 SD below the mean of healthy persons aged 20–30 years of the same ethnic group	x		Gait speed: ≤1.0 m/s or Walking distance < 400 m during a 6-min walk
Foundation of NIH Sarcopenia Project [11]	Weakness and low lean mass	ALM_BMI_	Grip strength		x
		- Men: <0.789	- Men: <26 kg		
		- Women: <0.512	- Women: <16 kg		
	Slowness with weakness and low lean mass			AND	Gait speed: ≤0.8 m/s

ASM/ ht^2^ = ratio of appendicular skeletal muscle mass over height squared; ALM/ht^2^ = ratio of appendicular lean mass over height squared; ALM_BMI_ = ratio of appendicular lean mass over body mass index; SD standard deviation.

A wide range of techniques can be used to measure the different components of sarcopenia [12]. Three techniques can be used for the measurement of appendicular lean mass: body imaging techniques, bio impedance analysis and anthropometry measures. In research, the two gold standards are the computed tomography (CT-scan) and the magnetic resonance imaging (MRI). However, because of the high costs and the limited access to this kind of equipment, the European Working Group on Sarcopenia in Older People (EWGSOP) [8] recommends in clinical practice, first the use of either dual energy X-ray absorptiometry (DXA) or, as a portable alternative to DXA, the bioelectrical impedance analysis (BIA). Despite their easy use in clinical practice, the anthropometric measures are not recommended for the diagnosis of sarcopenia because these measures are not validated in older people and are, therefore, vulnerable to error. Several techniques are also available for the measurement of muscle strength. Three techniques could potentially be used for the diagnosis of sarcopenia: handgrip strength, knee flexion or knee extension strength and the measurement of peak expiratory flow. In clinical research, the handgrip strength is the most widespread method. Indeed, this method does not require any special equipment, has been documented as a good marker of physical performance among community-dwelling older people and is well correlated with leg strength [13, 14]. Finally, the physical performance can be measured by the “short physical performance battery test (SPPB)”, by the “usual gait speed” or by the “timed up and go test” or “stair climb power test”. The EWGSOP [8] recommends the use of either the usual gait speed, measured on a 4-meter distance or the SPPB test [15] which is a composite measuring walk speed, balance and the ability to stand up 5 times from a chair. Different cut-offs have been developed by the EWGSOP for each variable and could be applied for the diagnosis of sarcopenia. Recently, the Foundation of NIH Sarcopenia Project proposed recommendations for cut-off points for weakness and low lean mass definitions aiming to provide an operational definition for sarcopenia. It was recommended to assess muscle strength by grip strength with cutpoints <26 kg in men and <16 kg in women, and low lean mass by appendicular lean mass adjusted to BMI, with respective cutpoints <0.789 kg/m^2^ and <0.512 kg/m^2^[16].

Given the variability in the definitions of sarcopenia, it is still a challenge to establish the actual prevalence of sarcopenia according to age and gender and to assess the direct and indirect impacts of sarcopenia on public health. The aim of this review is to discuss, both broadly and specifically, the public health implication of sarcopenia and its association with objectives health-related outcomes such as falls, fractures, admission in nursing homes or mortality.

## Discussion

### Epidemiology of sarcopenia

Sarcopenia is very common in older people. Currently it is still a public health challenge to establish a prevalence of sarcopenia. Indeed, this estimated prevalence depends on the type of studied population. A large number of studies have assessed the prevalence of sarcopenia within a cohort of adult subjects and this estimated prevalence could range from 0.1% to 85.4% according to patients’ characteristics [17–22]. Globally, a higher prevalence of sarcopenia is often observed in men, in elderly subjects, in subjects living in nursing home, in subjects having a low body mass index but also in subjects having a low educational level. The prevalence of sarcopenia seems also to differ according to ethnicity. Indeed, a higher prevalence of sarcopenia is observed in Asian people and a lower prevalence is observed in people with dark skin compared to Caucasian people. Recently, a systematic review [23] on the prevalence of sarcopenia has been published. It indicates that the prevalence of EWGSOP-defined sarcopenia is 1-29% for older adults living in community. The differences in prevalence seem attributable to the age of the population and the methods of assessment used but also to the cut-offs used for the diagnosis.

Prevalence of sarcopenia could also differ depending on the definitions used for the diagnosis of sarcopenia, as recently highlighted in the comparison of the FNIH criteria with the International Working Group and the European Working Group for Sarcopenia in Older Persons [11]. In 2013, Batsis et al. [24] compared eight definitions of sarcopenia and found a prevalence ranging from 4.4% to 94% across definitions. In 2013, Bijlsma et al. found that the prevalence of sarcopenia with different diagnostic criteria ranged from 0% to 20.8% in the lowest age category (below 60 years), from 0% to 31.2% in the middle (60 to 69 years) and from 0% to 45.2% in the highest (above 70 years) [25]. As expected, studies using muscle mass as single criterion of diagnosis revealed a higher prevalence of sarcopenia than studies based on the EWGSOP consensus algorithm. The choice of cut-off limits applied could also influence the prevalence of sarcopenia. This is confirmed in a study (performed in our Department, in press) showing that the prevalence of sarcopenia can vary from 9.25% to 18% depending on the cut-offs used. This same study also shows the importance of the diagnostic tool chosen for the measurement of muscle mass, muscle strength and physical performance. Depending on the tool used, the prevalence of sarcopenia can range from 8.4% to 27.6%.

Sarcopenia is also often related to multiple pathologies and comorbidities which can also compromise the measurement of its prevalence. Some authors are actually interested in sarcopenia in combination with another health issue, like osteoporosis, osteopenia, obesity, type II diabetes mellitus, breast cancer, etc. The prevalence of sarcopenia is systematically higher in subjects presenting another health condition than in healthy subjects. Sarcopenia could be, in this case, considered as one consequence of this health problem.

This confused state and the current impossibility of establishing a clear prevalence of sarcopenia makes comparisons between studies difficult and thus represents an important public health issue. Moreover, the various values for the prevalence of sarcopenia found across studies are probably associated with different characteristics of sarcopenic subjects which could compromise the implementation of pertinent therapeutic strategies in the field of sarcopenia.

### Consequences of sarcopenia: Indirect impact on public health

Many consequences of sarcopenia are prognostic indicators of public health burden, such as the development of physical disability, nursing home admission, depression, hospitalization, and even mortality [26]. In particular, sarcopenia is associated with poor physical performance, functional decline and physical disability [22, 26]. Sarcopenia predicts loss of independence for daily life activities in elderly men and women [27, 28], and also affects gait speed or regularity. Leg lean mass has been identified as an independent predictor of the level of mobility impairment assessed by the SPPB test [29]. Ability to walk is an obvious determinant of subsequent disability, mortality, and health care costs [30]. Sarcopenia is also associated with falls, a well known issue regarding the risk of fracture and disabilities (odds ratio for fall in the sarcopenia group relative to the normal group: 4.42 (95% CI 2.08-9.39) in men and 2.34 (95% CI 1.39-3.94) in women) [31].

Sarcopenia is also associated with many comorbidities which have a major impact on public health. As occurring concomitantly with age-related bone loss, sarcopenia coexists with osteoporosis and may increase fracture risk, potentially directly via crosstalk between muscle and bone tissues [32, 33] and indirectly via increase of risk of falling [34, 35]. Most of endocrine diseases (diabetes, hypogonadism, hypercortisolism…) as well as obesity, or chronic kidney disease [34], are associated with sarcopenia independently of age-related muscle loss, which may be an underlying mechanism by which chronic diseases cause physical disability [36].

In this context, sarcopenia is also associated with greater risk of hospitalization [37] and is highly prevalent among older adults admitted to acute care wards [38] or in nursing homes [39]. Sarcopenia is also a predictor of bad outcomes in patients who undergo major general or vascular surgery [40] or with serious illness, such as in transplantation or cancer outcome [41, 42]. All these health-related consequences of sarcopenia are supposed to alter quality of life in these patients [43].

**Figure 1:**
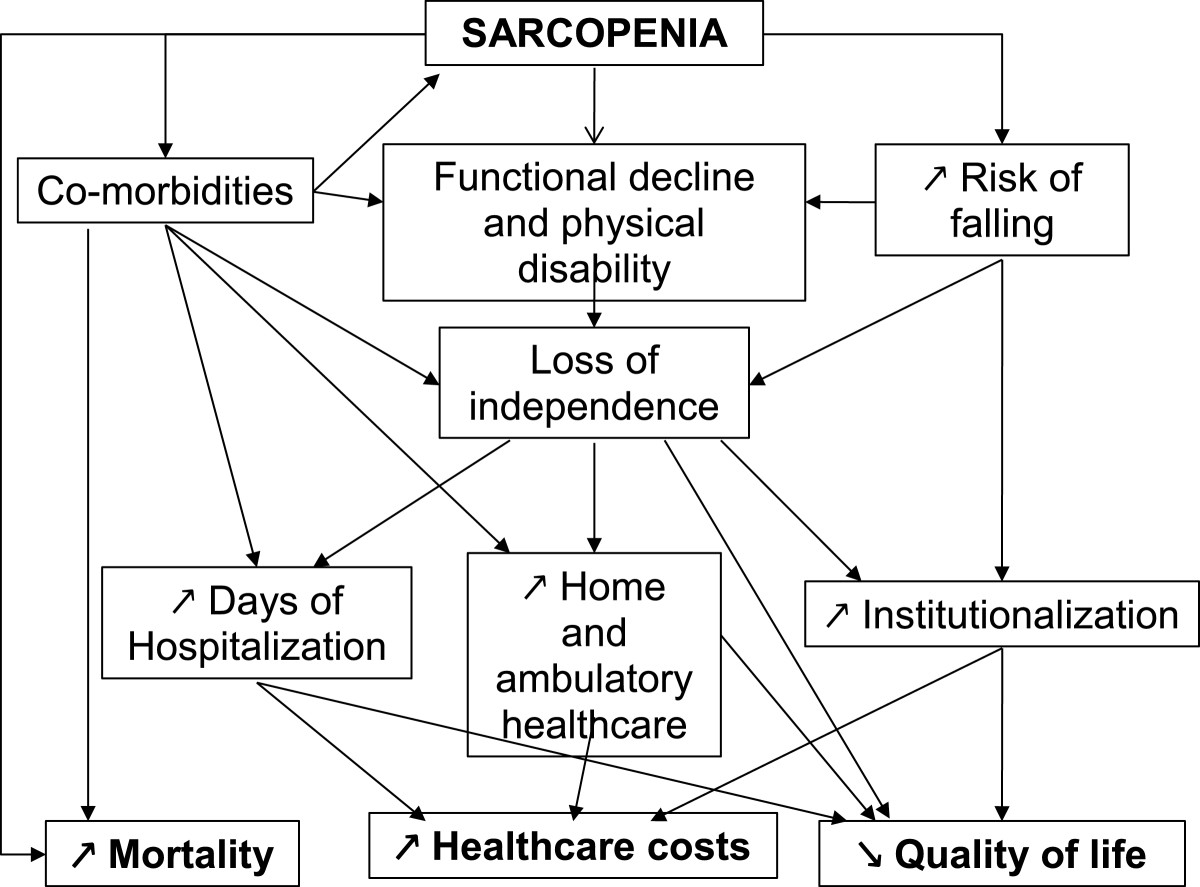
**The complex burden of sarcopenia on public health.**

Importantly, several studies indicate that sarcopenia and indicators of alterations of muscle strength (such as grip strength, walking speed, chair rises, or standing balance) predict future mortality in middle-aged and older adults [21, 44]. Sarcopenia is also associated with short- and long-term mortality in hospitalized patients [38], or in nursing home elderly residents [45].Taken together, these data highlight how sarcopenia may impact various public health components, at the patient level with higher rate of disabilities, loss of independence, bad comorbidities outcome, institutionalization or mortality, but also at the societal level, contributing to major healthcare and dependence costs in disabled sarcopenic elderly (Figure 1). However, none of the proposed operational definitions of sarcopenia demonstrated its superiority to be predictive of these health-related “hard” outcomes, such as fractures, falls, admission in nursing homes, or mortality. Future researches are clearly needed in this field to clarify which operational definition of sarcopenia should be integrated in clinical practice to diagnose and target sarcopenia and its impact on public health.

### Public health costs of sarcopenia

Disability is associated with an increased risk of hospitalization and nursing home placement, increased home healthcare and, obviously, health care expenditure. Given the effect of sarcopenia on disability, public health costs of sarcopenia are expected to be high. Currently, economic data on sarcopenia are very poor. Only one study has currently reported the healthcare costs of sarcopenia in the United States [46]. Those estimates have taken into consideration the direct costs of sarcopenia which raised, in 2000, to $18.5 billion, $10.8 billion in men and $7.7 billion in women. These costs are represented by hospitalization, nursing home admissions and home healthcare expenditure. In 2000, this amount represented about 1.5% of total health expenditure in the United States. It must be added that, in addition to disability, sarcopenia is associated with multiple comorbidities and may also have effect on osteoporosis [47], obesity [48] and type II diabetes mellitus [49]. Whith these comorbidities associated healthcare costs taken into account, the economic burden of sarcopenia may probably be even more important than reported in the study of Janssen [46]. This study is currently unique and, until now, no reliable economic assessment of sarcopenia has been performed in Europe.

Despite this lack of other economical assessment, several studies have however looked at the relationship between sarcopenia and different area of expenditure such as hospitalization or nursing home admission. In the United kingdom, one study has shown that, in comparison with patients without sarcopenia, those diagnosed with sarcopenia presented a mean length stay in hospital significantly higher (mean of 13.4 ± 8.8 days for sarcopenic subjects versus 9.4 ± 7 days for non-sarcopenic subjects; p = 0.003) [50]. The association between sarcopenia and hospitalization was examined in another study [37] showing a significant association between low muscle density (RR 1.5, 95% CI 1.2-1.7) and grip strength (RR 1.5, 95% CI 1.3-1.8) with hospitalization. Lean mass was however not associated with risk of hospitalization.

Although some studies have shown a higher risk of institutionalization among frail people [51–53], regarding sarcopenia specifically, no study has currently assessed the relationship between sarcopenia and nursing home admissions [54].

Sarcopenia is also associated with other healthcare costs area such as loss of productivity, reduced quality of live and loss of autonomy but also with psychological problems. However, these indirect costs of sarcopenia have never been quantified, neither in the US, nor in Europe.

In their assessment of healthcare costs of sarcopenia in the United States, Janssen et al. [46] also examined the effect that reduced prevalence of sarcopenia would have on healthcare expenditure, through for example pharmacological treatment, public health campaigns, physical activity intervention,. They found that a 10% reduction in the prevalence of sarcopenia would result in saving $1.1 per year in the US. In a public health context, this potential economic saving is important. In comparison with osteoporotic fractures, for which the economic costs are similar [55] and for which numerous public health campaigns are organized aiming at reducing their occurrence, it is startling to note that, for sarcopenia, no public health campaigns are directly aimed at reducing the prevalence of this important geriatric syndrome. Because the number of older people is increasing all over the world, health policy decision-makers should consider some money investment in sarcopenia prevention and treatment to ensure important future savings.

### Targeting sarcopenia: potential impact on public health

Obviously there is currently no consensual operational definition of sarcopenia. This age-related condition has numerous consequences in public health, illustrated with relevant hard clinical outcomes such as falls, fractures, hospitalisations, institutionalizations, mortality. These consequences directly induce high personal, social and health care systems costs, which will most certainly increase steadily with population ageing. The implementation of effective and broadly applicable preventive interventions has become a medical and societal challenge for the growing number of older persons affected by sarcopenia and its disabling complications. Identifying and targeting the determinants of sarcopenia is a necessary first step to limit its impact on public health (Figure 1). In addition to the identification of the determinants of skeletal muscle loss, research strategies will have to include a lifecourse approach focused on factors associated with peak muscle mass and strength, such as birth weight [56] and early nutrition [57]. Nutritional interventions may influence sarcopenia, in particular diets rich in proteins and antioxidant nutrients, as well as vitamin D or omega-3 fatty acids supplements. Various exercise-related interventions (resistance exercise training, gait, balance, coordination and functional exercises) have been tested, targeting muscle strength, physical function, the risk of falls and balance in older people [58]. Potent pharmaceutical therapies have been proposed, such as hormone therapies (growth hormone, testosterone, selective androgen receptor modulator dehydroepiandrosterone, estrogen), angiotensin converting enzyme inhibitors, ghrelin agonists, but with up to now, little convincing effects or with presenting adverse side effects [58]. One of the most promising approaches may be the inhibition of myostatin, a regulator of muscle development and growth [59, 60]. It is likely that combining lifestyle, nutritional, pharmacological and physical interventions is the most promising strategy. Clinical trials are currently conducted in this direction, such as the DoHealth study, which combines vitamin D, omega-3 fatty acids and physical exercise for the prevention of diseases at older age (ClinicalTrials.gov Identifier: NCT01745263). The cost-benefit ratio of these interventions will have to be assessed in health economic models based on health care utilization and incidence of chronic diseases. However, a gap persists regarding assessment of specific health conditions related to sarcopenia, as fracture has become the relevant outcome to evaluate interventions targeting osteoporosis. Validation of specific, objective and reproducible outcomes or tools is a necessary step before considering the development of interventions targeting sarcopenia and likely to be recognized both by the scientific and medical community and regulatory agencies.

## Conclusion

Sarcopenia has become a major health condition associated with ageing, and contributes to many components of public health at both the patient and the societal levels. It interferes with the incidence and prognosis of many comorbidities, and obviously increases health care utilization. It is a determinant of loss of independence, leading to institutionalizations or prolonged hospitalizations. All these aspects increase healthcare costs for the society, and affect quality of life and mortality of sarcopenic patients. With the improvement of life expectancy and the consensual previsions of marked increase of the proportion of older people, it is urgent to consider the economic and societal burden of sarcopenia, and to implement interventions to prevent and treat sarcopenia in the ageing population.

## Authors’ original submitted files for images

Below are the links to the authors’ original submitted files for images. Authors’ original file for figure 1

## Abbreviations

ALM: Appendicular lean mass
MRI: Magnetic resonance imaging
EWGSOP: European working group on sarcopenia in older people
DXA: Dual energy X-ray absorptiometry
BIA: Bioelectrical impedance analysis
SPPB: Short physical performance battery.
