# Supplementary figures and images for: Sarcopenia: burden and challenges for public health
Source: Arch Public Health. 2014 Dec 18;72:45. doi: 10.1186/2049-3258-72-45 (PMC4373245; doi:10.1186/2049-3258-72-45)

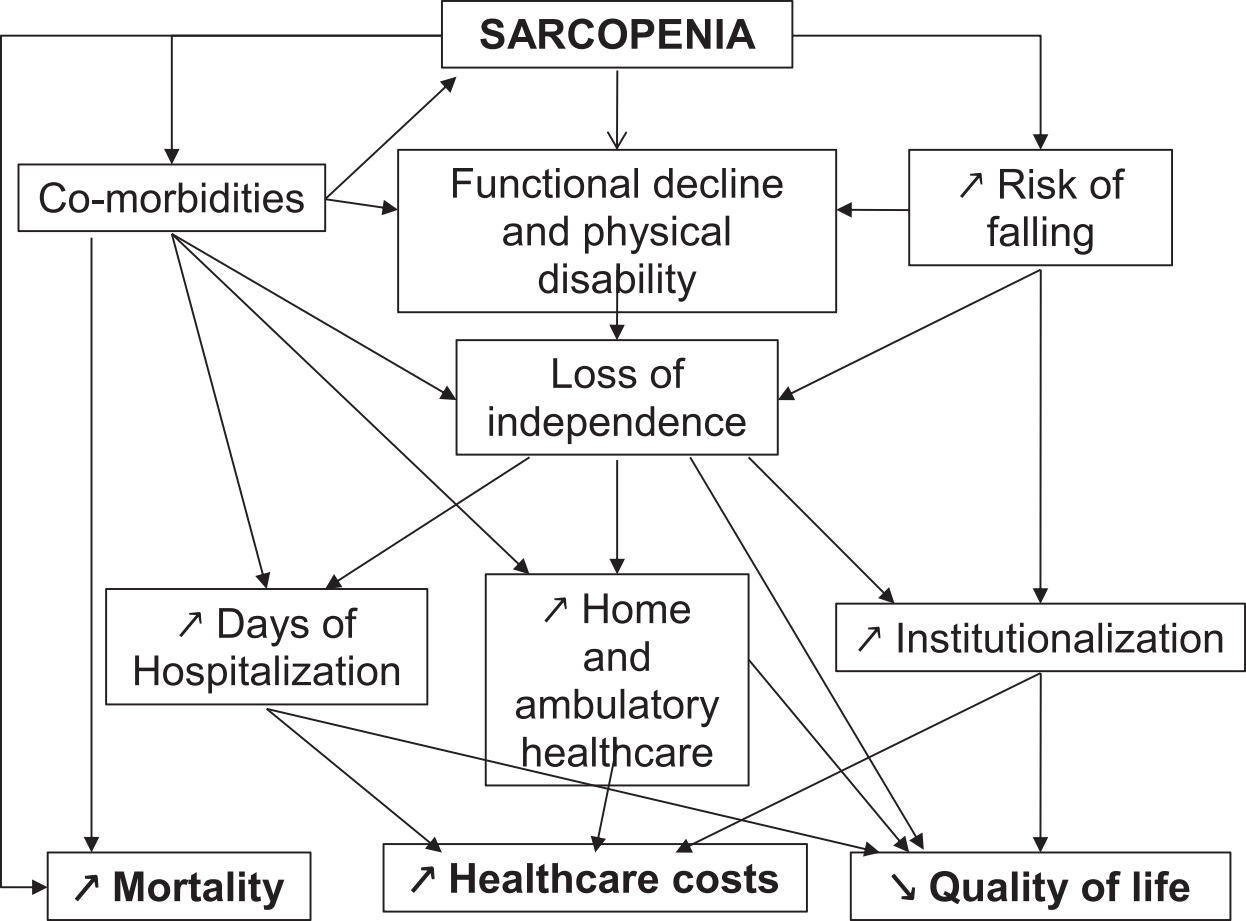

Supplement: Supplementary file 1 — Authors’ original file for figure 1 [file 13690_2014_5064_MOESM1_ESM.pdf]
